# The Impact of Flavonoid-Loaded Nanoparticles in the UV Protection and Safety Profile of Topical Sunscreens

**DOI:** 10.3390/biom13030493

**Published:** 2023-03-07

**Authors:** Magda Fonseca, Mubashar Rehman, Raquel Soares, Pedro Fonte

**Affiliations:** 1 EPI Unit, Department of Epidemiological Research, Institute of Public Health of University of Porto (ISPUP), Rua das Taipas 135, 4050-600 Porto, Portugal; 2 Department of Pharmacy, Quaid-i-Azam University, Islamabad 45320, Pakistan; 3 Department of Biomedicine, Faculty of Medicine, University of Porto, Al Prof Hernani Monteiro, 4200-319 Porto, Portugal; 4 I3S, Instituto de Investigação e Inovação em Saúde, Universidade do Porto, Rua Alfredo Allen, 208, 4200-135 Porto, Portugal; 5 Center for Marine Sciences (CCMAR), Gambelas Campus, University of Algarve, 8005-139 Faro, Portugal; 6 Department of Chemistry and Pharmacy, Faculty of Sciences and Technology, Gambelas Campus, University of Algarve, 8005-139 Faro, Portugal; 7 IBB—Institute for Bioengineering and Biosciences, Department of Bioengineering, Instituto Superior Técnico, Universidade de Lisboa, 1049-001 Lisboa, Portugal; 8 Associate Laboratory i4HB—Institute for Health and Bioeconomy at Instituto Superior Técnico, Universidade de Lisboa, Av. Rovisco Pais, 1049-001 Lisboa, Portugal

**Keywords:** sunscreen, UVA photoprotection, UV filter, antioxidant, flavonoid, nanoparticle, skin cancer

## Abstract

Excessive UV radiation exposure is harmful to skin cells since sunburn is accompanied by oxidative burst, leading to a rapid increase in skin cancer. However, the insufficient UV photoprotection of approved sunscreens and the negative impact of their compositions on ecosystems and human health makes the utility of sunscreen a questionable recommendation. Therefore, discovering UV filters with significant antioxidant activity and improved topical performance and photostability is an urgent need. Recently, the use of nanosized natural molecules incorporated in sunscreens has been a scientific hot topic, as it has been suggested that they provide a synergistic effect with synthetic UV filters, improving overall SPF and antioxidant activity, higher retention on the epidermis, and less toxicity. The aim of this review was to verify the usefulness of sunscreens incorporating flavonoid-loaded nanoparticles. A literature review was performed, where original and review articles published in the last 6 years were analyzed. Formulations containing nanosized flavonoids with improved UVA photoprotection and safer toxicological profiles, associated or not with synthetic filters, are promising sunscreens and more clinical investigation must be performed to validate these findings.

## 1. Introduction

The sun is considered the source of life, but due to the diminished stratospheric ozone layer, the sun’s harmful UV (ultraviolet) light reaches the atmosphere and penetrates exposed human skin, causing considerable damage to skin cells if the endogenous repair system is not prepared to fight the accumulated damage. Oxidative burst, pro-inflammatory cascade, photosensitization, and photoirritation of the skin are the main adverse consequences of the insufficient response of the cutaneous endogenous antioxidant system to excessive UV exposure, namely, to ultraviolet A (UVA) radiation (the type of UV rays that interact with proteins on the dermis, being responsible for the oxidative burst events) [1,2,3,4].

To protect against this process, there are topical sunscreens which form a protective film against UV radiation on the surface of the skin epidermis. Sunscreens contain UV filters which are, usually, synthetic organic or inorganic molecules that can absorb, reflect, or scatter UV rays, according to their chemical nature. These cosmetic formulations must be photostable and not toxic to human skin cells [4,5,6,7].

Although the sales of approved sunscreens have been increasing worldwide, negative effects of UV radiation on the skin, such as pigmentation, aging, and cancer, have been gradually observed, suggesting that the current conventional sunscreen products are not as successful as they should be [8]. Some studies have shown that synthetic UV filters, known as the conventional ones, can cause oxidative stress phenomena on skin cells if they are photoinstable, adding extra oxidative damage to the one naturally caused by UV exposure, as already mentioned. It is suspected that these additional oxidative events might be the major reason for the current gradual increment in skin cancer, for instance. Another disadvantage of conventional UV filters is the harmful effect on ecosystems, leading to their disruption, which negatively impacts the human body [9,10,11,12].

Therefore, it has become mandatory to investigate new, safer, and more efficient UV filters and sunscreen formulations overall, that are more cosmetically attractive and less toxic to human skin. A promising alternative are natural UV filters, extracted from natural sources or chemically synthetized from plants, fruits, or other natural ubiquitous derivatives, with structural modifications that increase their efficacy as photoprotectors and free radical scavengers, and decrease their ecological and human toxicity. Flavonoids are a type of natural UV filter that have been attracting interest from the scientific community and some promising results have been published recently [13,14]. Because flavonoids, as other natural molecules, do not have the best physicochemical properties to permeate the skin in an efficient way, researchers have been studying the possibility of delivering them to the skin surface through nanocarriers incorporated in traditional sunscreen formulations as creams or lotions [15,16]. Lipid-based nanoparticles, polymeric nano vehicles or gold nanoparticles are the main nano transporters selected to encapsulate flavonoids with UV protection capacity, and the results obtained so far are encouraging [17]. Accordingly, current research has suggested that flavonoids loaded in nanosized carriers are possible new sunscreen formulations with a higher potential for UVA photoprotection, as well as an improved safety profile for ecosystems and human health, in comparison with conventional synthetic UV non-nanosized formulations. So far, the scientific community has mainly directed its efforts towards the study of bioactives with UVB protection, which led us to consider research related to UVA natural filters as a new and innovative topic. In addition, flavonoids are ubiquitous in nature; therefore, it is expected that incorporating flavonoids into topical nano sunscreens may produce more sustainable and ecological formulations, which is a mandatory milestone trend in the pharmaceutical and cosmetic industries.

The aim of this review was to answer the following question: what is the attributable utility of nanoformulations containing flavonoids on the overall photoprotection of a topical sunscreen, concerning flavonoids’ synergistic UVA photoprotective activity, and on its toxicity profile towards ecosystems and human health? The literature published over the last 6 years was searched in MEDLINE (PubMed), and original research and review articles were analyzed for relevant and promising results.

## 2. Structure and Effect of UV Radiation on Skin

**Figure 1:**
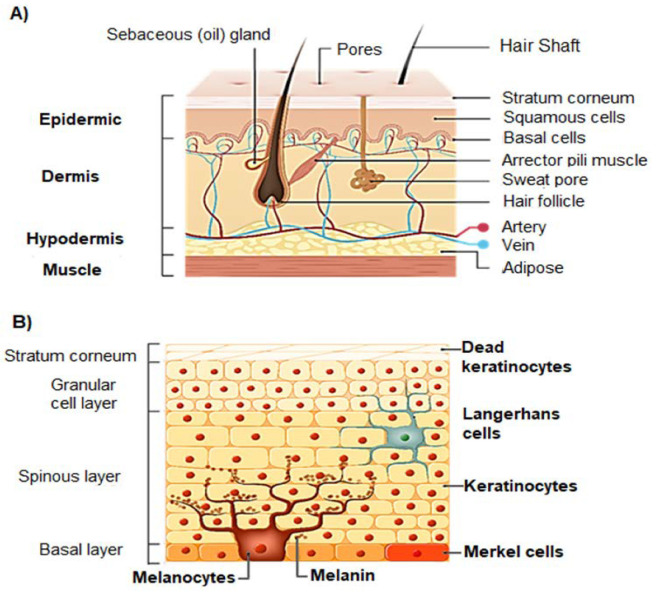
Structure of human skin (**A**) and structure of the epidermis (**B**).

Briefly, the skin, the largest organ of the human body, is composed of three main layers: epidermis, dermis, and hypodermis (Figure 1A). The epidermis consists of the stratified squamous epithelium. Starting from the deepest layer of skin towards the surface, there is the basal stratum, the spinous stratum, the granular stratum, the lucid stratum (only present in thick skin), and the stratum corneum (Figure 1B) [18]. The cells of the epidermis are constantly renewed, from their junction with the dermis to the skin surface, where a permanent peeling occurs [19]. The epidermis has four types of cells, namely, Langerhans cells, keratinocytes, Merkel cells, and melanocytes (Figure 1B) [18]. The stratum corneum has a thickness between 10 to 15 μm. It acts as semi-permeable barrier to the penetration of compounds and UV radiation, and it is the most effective barrier. The stratum corneum is made of corneocytes that are terminally differentiated keratinocytes embedded in a highly ordered hydrophobic matrix composed of ceramides, cholesterol and cholesterol esters, and fatty acids. The other sublayers of the epidermis contribute to the observed thickness of 50 to 100 μm [20].

The epidermis, especially the stratum corneum, seems, therefore, to be the rate limiting step for a nanoparticulate flavonoid system delivery through topical administration, as is the case of topical natural sunscreens.

**Figure 2:**
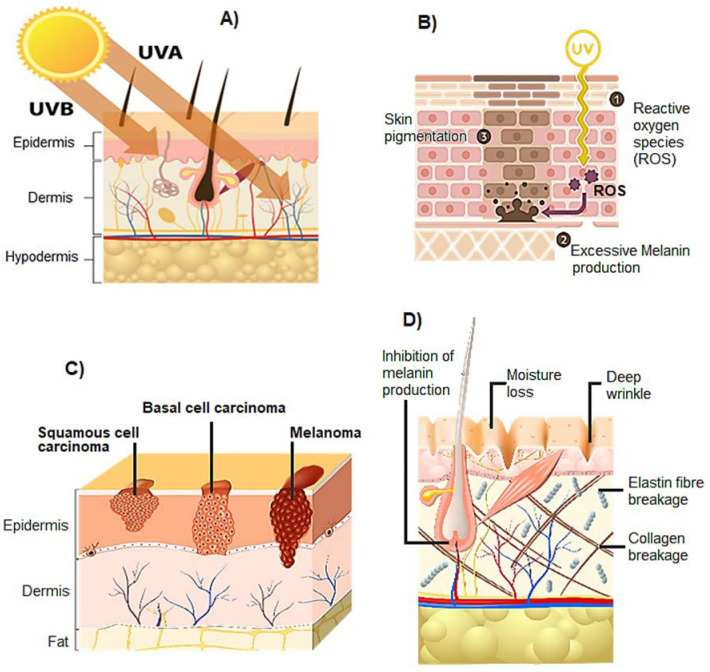
Skin permeation capacity of UVA and UVB radiation, and three main consequences of repeated exposure to UV radiation on the normal physiology of skin: (**A**) UVA light penetrates the dermis, while UVB radiation is absorbed by the epidermis; (**B**) hyperpigmentation; (**C**) carcinogenesis; and (**D**) aging.

Most incoming UV radiation (90–95%) belongs to the UVA (315–400 nm) waveband. Ultraviolet B (UVB) radiation lies in the range of 290–315 nm and is absorbed mostly in the epidermis. UVA, on the other hand, penetrates deeper into the skin and affects the epidermis, the dermis, and their principal cells: keratinocytes, melanocytes, and fibroblasts (Figure 2A) [1]. UVA radiation causes the generation of higher levels of ROS (reactive oxygen species) and RNS (reactive nitrogen species) through the interaction with endogenous chromophores. Repeated exposure to UV radiation causes the gradual accumulation of damaged molecules, resulting in skin hyperpigmentation (Figure 2B) [5,6,7], cancer (Figure 2C) [21,22], aging (Figure 2D) [23,24], erythema [25,26,27,28,29], and immune suppression [30,31].

Among the most significant biochemical effects of UV radiation are DNA (deoxyribonucleic acid) damage [32,33], protein oxidation [34], and lipid peroxidation [35,36]. DNA and RNA (ribonucleic acid) are the main targets of UVB radiation. Pure DNA, RNA, proteins, or lipids do not absorb UVA radiation, whereas the complexes formed by DNA with transition metals, such as iron, absorb slightly in the UVA range. This phenomenon is responsible for the nicking of DNA by UVA in the presence of iron and oxygen [37]. The nucleic acids and protein damage caused by UVB radiation is the reason UVB filters are the main type of UV filters present in sunscreens. An interesting relationship between the high concentration of nucleic acids and proteins in the epidermis and the absence of the penetration of UVB rays in the dermis can be established.

Direct absorption of UVB by proteins does not induce stereochemical modifications. Protein modifications are the consequences of photosensitized oxidation. In opposition, modifications to the bases of nucleic acids occur when they absorb UVB radiation. Adjacent pyrimidines can react and form photoproducts, the most frequent being CBD (cyclo-butane dimmers) and pyrimidine dimmers, e.g., pyrimidine 6-4 primidone. CBD and pyrimidine dimers induce stereochemical changes in the structure of nucleic acids. The removal of pyrimidine dimers and of other nucleic acid damage requires the removal of the short nucleic acid segment that contains the damage and the re-synthesis of the short sequence. This process is not free of errors, and it is generally regarded as the major cause of the mutagenicity and carcinogenicity of UV radiation [34].

Pyrimidine dimers in DNA have been shown to trigger an inflammatory reaction [38]. The inflammatory reaction is accomplished by the proteolytic cleavage of the elastic fibers, performed by the matrix metalloproteinases which are secreted by fibroblasts upon the release of cytokines by the immune cells, while these immune cells are chemotactically driven across the dermis to reach the UV-damaged cells [2]. The inflammatory reaction is also accomplished by the oxidative stress induced by hydrogen peroxide released by immune cells when crossing the walls of the blood vessels to enter the dermis to digest the UV-damaged cells. During this phagocytic process, more hydrogen peroxide is released. There is also a release of singlet oxygen when the immune cells cross the dermis and aggravate the oxidative stress [3].

Photosensitization is characterized by the production of several other ROS. Tyrosine and tryptophan exposed to UV generate hydrogen peroxide which, in the presence of transition metals, is converted in the hydroxyl radical [39]. In addition, several photosensitizers transfer charge to molecular oxygen to form superoxide and singlet oxygen [40,41]. All these ROS can trigger the peroxidative cascade in lipids and damage cell membranes [35,36]. Lipid peroxidation is one of the most recognized biochemical events triggered on an inflammatory cascade.

A key phenomenon that can be observed in cultured cells exposed to UV rays is the onset of blebbing [42]. Blebbing is the appearance of bubbles on the surface of cells, and it is one of the cellular responses to stress and damage. It is a morphological phenomenon caused by oxidative stress [43] that can be analyzed by microscopical and ultrastructural technologies [44]. Another effect of UV exposure in cultured cells is the fragmentation of chromatin which is a specific indicator of apoptosis or programmed cell death [45]. Apoptosis is also accompanied by the blebbing phenomenon.

Often neglected by the skin care industry, nitric oxide (NO) is a vasodilating agent generated by inducible and constitutive nitric oxide synthase within cells. NO participates in sensitization events such as cutaneous itch. Inducible NO synthase produces NO as a defense mechanism in response to cytokines that are secreted upon infection or other aggressions. UVB exposure induces the expression of inducible NO synthase in vessel endothelia of normal human skin and in cultured human dermal endothelial cells, and exposure to UVA, in the absence of cytokines, increases the expression of nitric oxide synthase-2 in human skin organ cultures [46].

## 3. Sunscreens and UV Protection

### 3.1. Sunscreens

The topical use of sunscreens represents the most popular strategy of skin photoprotection [47] in response to the depletion of ozone in the stratosphere and the consequent higher accumulation of UV radiation on the surface of our planet.

**Figure 3:**
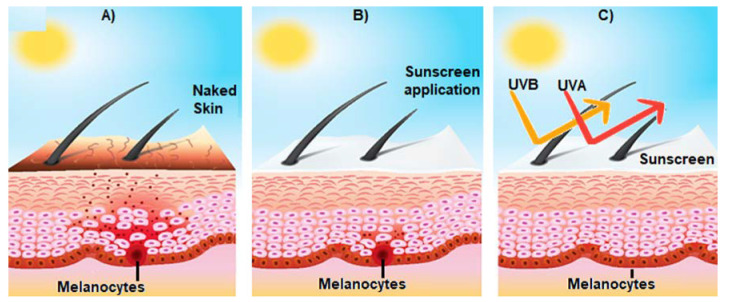
Effect of sunscreen on the prevention of sun-induced damage. (**A**) Shows the activation of epidermal melanocytes by sun UV radiation and the consequent release of melanin (brown spots) towards the surface of the skin, giving it a brown tone; (**B**) represents the consequences of the use of sunscreen on skin structure; and (**C**) broad spectrum sunscreen prevents the damage of the skin induced by UVA and UVB radiation.

An ideal sunscreen should have (a) a technology to reduce the intensity of UV rays reaching the surface of the skin, and (b) a technology to prevent or reduce the oxidative burden caused by the release of free oxygen and nitrogen reactive species upon UV radiation absorption, namely, appropriate antioxidants and scavengers of free radicals, or inhibitors of their formation. This is particularly important because singlet oxygen is formed not only by endogenous photosensitizers but many commercial sunscreens containing titanium dioxide (TiO_2_) and zinc oxide (ZnO) are also reported to produce ROS [9] (Figure 3).

Besides the mentioned requirements, an ideal sunscreen should also: (a) absorb a broad range of UV rays; (b) not be chemically broken down to prevent a decrease in efficacy or an increased toxicity or irritation due to the by-products; (c) have suitable properties while formulated as a cosmetic base and penetrate the skin easily; (d) not get removed by water or perspiration; (e) avoid the need of frequent reapplication; (f) be effective, at low concentration; and (g) not cause irritation, sensitization, and toxicity to the skin [5].

Permeation of sunscreen components through the skin is a fundamental parameter to be considered when formulating the composition of a sunscreen. A sunscreen should remain at the surface of the skin and no absorption should occur so that it can perform its function without causing toxicity [48]. As referred above, sun filters should not undergo modification when exposed to UV radiation, therefore, the photostability of the sunscreen also becomes mandatory. If the UV filters are not photostable enough, they become less absorptive and their function of protecting the skin is therefore lost [49].

The efficacy of a sunscreen is usually measured by the Sun Protection Factor (SPF) which corresponds to the number of times the amount of 2 mg/cm^2^ of sunscreen application (standardized amount) increases its capacity of delaying the formation of erythema due to sun exposure, compared with unprotected skin. SPF is also known as a measure of how much more sun exposure it takes to undergo sunburn. For example, a sunscreen with an SPF of 30 means that it will take 30 times more sun exposure to develop erythema when compared with the same skin without photoprotection [50]. Therefore, sunscreens are mostly classified according to their SPF which can reach up to 50 in Europe [51] and should be, at least, 30. It is assumed that this range of SPF values is adequate to provide enough daily photoprotection against UVB radiation. In theory, when applied evenly on skin at 2 mg/cm^2^, SPF 50 sunscreen should filter out 98% of UVB rays and lengthen the time it takes for the skin of a person to redden in the sun [52,53]. Accordingly, SPF does not consider protection against UVA radiation, even though many current sunscreens have UV filters for both UV rays. Nevertheless, the protection against UVA rays tends to be inferior. Although there are European standards for UVA protection, which recommend that the UVA Protection Factor (PFUVA) should be, at least, one third of the marketed SPF, many of the current marketed sunscreens do not meet the requirement mentioned above. 

Sunscreens should be applied at least 15 min before going outside. It is important to use a sun protective lip balm, as well. For a correct use of a sunscreen, it should be reapplied every 2 h and after swimming or excessive sweating, to provide a sustained skin photoprotection. Many cosmetics contain UV filters, which are beneficial for the product but insufficient to provide adequate protection against both UVA and UVB radiation. Applying a sunscreen after the usual cosmetics products should be a daily habit, throughout the year [12]. It is already demonstrated that sunscreens inhibit sunburn because they suppress the mechanisms that cause erythema. However, it is still unclear if they prevent the underlying biochemical processes. 

A person may not get sunburned, but still have several unwanted effects occurring in the skin. The UV filters oxybenzone, octocrylene, octinoxate, PABA (para-aminobenzoic acid), and 4–methylbenzyliden camphor have been reported to induce free radicals [25], induce caspase enzymes linked with photosensitization [26], stimulate melanoma tumor growth [27], and neurotoxicity [1,27,28,29,54]. A very damaging free radical, singlet oxygen, is formed by commercial sunscreens containing TiO_2_ and ZnO [9], as mentioned earlier. Therefore, it is suggested that the elimination of sunburn by sunscreen use is not free of toxic effects which can lead to the future development of skin cancers and other types of toxicities [55]. 

Based on published scientific data, sunscreens do not prevent skin cancers associated with intentional sun exposure. Therefore, the risks associated with intentional sun exposure are outweighed by the lack of benefits. In addition to the use of sunscreen, people who want exposure to sun light are advised to avoid peak hours of UV radiation (10 a.m.–4 p.m.), wear protective clothing including a broad-brimmed hat with sunglasses, and/or use an oversized umbrella/cabana when at the beach or pool. The practice of all these healthy sun habits will significantly help prevent the development of skin cancers [12].

### 3.2. Conventional UV Filters

#### 3.2.1. Organic Filters

Organic filters absorb specific wavelengths of UV radiation depending on their chemical structure. The ground state of low energy is converted into a high-energy level. Organic filters are divided into three types based on how they process the high levels. First, the photostable filter dissipates the absorbed energy as heat energy to the atmosphere, returning it to a low-energy level. They are efficient at reabsorbing UV energy. Photo-unstable filters, upon absorption of UV energy, undergo a change in their chemical structure or degrade completely so they cannot absorb UV energy again. Photoreactive filters constitute the third type, and they interact with molecules in the microenvironment in their high energy or excited state. Photoreactive filters can react with proteins and lipids from skin cells and other ingredients from the sunscreen and surrounding oxygen; consequently, reactive oxygen and nitrogen species are generated and may lead to unwanted biological effects [5].

**Dibenzoylmethane derivatives**: They have a high absorption capacity in the UVA range, but they degrade in the presence of UV radiation, decreasing the efficiency of sun protection at the time of UV exposure. Photofragmentation of these filters occurs, leading to the formation of free radicals, which cause skin damage. Avobenzone is the most well-known derivative from this class [56].

**Benzophenone derivatives**: They absorb or dissipate UV radiation, mainly UVA. It was previously reported that cytotoxic effects are caused by these filters. Oxybenzone is an example of a UV filter [57].

**Para-Aminobenzoic acid and derivatives**: They absorb UVB radiation and can be retained for a long time in the surface of the skin. Photoallergic reactions are a common adverse reaction [58,59].

**Salicylate derivatives**: They are weak absorbers of UVB radiation and are used to minimize the photodegradation of other photo protectants. Homosalate belongs to this class [60].

**Benzotriazoles**: They can be photostable broad-spectrum filters, having an efficient sun protection ability. Due to their photostability, photoaging and photosensitization are less frequent, as well. Octrizole is a member of this class of UV filters [61].

#### 3.2.2. Inorganic Filters

Inorganic filters scatter and reflect UV radiation to the external environment. They function as a physical barrier to UV radiation. These filters are broad-spectrum as they can reflect the radiations in the entire UV range. The most recognized inorganic filters are TiO_2_ and ZnO [62].

Although the use of sunscreens has been increasing, the risk of development of skin cancer has also been increasing [8]. Sunscreens contain UVB filters to avoid sunburn and photoaging. Due to the insufficient ozone layer, UVA rays also reach the atmosphere and permeate the human skin epidermis into the dermis to induce an oxidative burden, which can cause carcinogenesis as the worst possible consequence, as discussed.

Interestingly, research suggests that most basal cell carcinomas may be primarily attributed to UVA irradiation [63,64]. UVA rays are absorbed by endogenous photosensitizers which subsequently cause oxidation reactions, producing reactive oxygen and nitrogen species [65,66,67,68]. Fortunately, endogenous antioxidant defense systems are present in the skin, including glutathione peroxidase, catalase, and superoxide dismutase, which protect the skin against oxidative damage [69]. However, when the production of reactive free radicals exceeds the capacity of endogenous antioxidant systems to protect the target cells, oxidative stress initiates, which has been associated with the occurrence of skin cancer [10].

UVA radiation can also penetrate window glass in buildings or cars, making sun protection a daily necessity, even in the winter season [12]. The value of the sunscreen ratio SPF/PFUVA or UVA/UVB absorbances must be analyzed, to ensure a homogeneous protection in the two UV ranges. Couteau et al. determined SPF, PFUVA, and UVA/UVB ratios of O/W (oil in water) creams formulated by the authors [70,71,72] with 22 organic filters (15 UVB filters, 4 UVA/UVB filters, and 3 UVA filters) and 2 inorganic filters, TiO_2_ and ZnO, in diverse combinations. The study of the blocking capacity in UVA and UVB range was determined using an in vitro method [70]. Each UVA filter was associated with TiO_2_ or ZnO used at 10%. Three associations with TiO_2_ resulted in an increase in the effectiveness of both the UVA and UVB ranges. Those conducted with butylmethoxydibenzoylmethane, anisotriazine, and diethylamino hydroxybenzoyl hexyl benzoate were the most successful. None of the combinations with ZnO presented a synergistic effect in both the UVA and UVB ranges. The combinations with TiO_2_ reach an SPF higher than 50, whereas combinations with ZnO led to a maximum SPF of approximately 39, as suggested in the results from anisotriazine assays [73]. These results suggested that the combinations of organic filters with TiO_2_ are preferable.

The human health risk associated with organic UV filters can be regarded as a concern because they can enter the body through percutaneous absorption and contaminated food and water consumption. The organic UV filters can reach the blood circulation and be found in body fluids such as urine, semen, and breast milk [4,6,7]. 

Despite the biological consequences of these substances being still undiscovered, it was reported that some marketed organic UV filters exhibit endocrine-disruption activity (Table 1) on the reproduction cycle of organisms [74,75]. In addition, the maternal transfer of organic UV filters in humans [76] and animals, such as dolphins [77] and birds [78], has been proven. Another reported effect of organic UV filters in the human organism is vitamin D deficiency, which can cause negative changes in bone metabolism and weaker immune responses [49].

Oxybenzone and other organic UV filters can induce photoallergic reactions and photocarcinogenic events, as described on Table 1, as well [12]. Concerning the inorganic UV filters, TiO_2_ and ZnO, the most well-known filters, they can block UV rays from coral algae and inhibit photosynthesis, subsequently, and may add to local increases in water temperatures, contributing to the devastating greenhouse effect [11].

### 3.3. Regulatory Considerations on Sunscreens

In February 2019, the U.S. FDA (Food and Drugs Administration) updated the regulatory requirements for non-prescription and over-the-counter sunscreens to ensure their safety, efficacy, and consistency in labelling. Broad-spectrum sunscreens are defined by the FDA as products that provide UVA and UVB protection at the usual ratio UVA/UVB of 1:3. They must have a minimum SPF of 30, be water-resistant, reduce the risk of development of skin cancer, decrease the incidence and severity of sunburn, and prevent photoaging [55].

UV filters can absorb, reflect, or scatter UV rays. Few UV filters used in FDA-approved sunscreens are considered as generally recognized as safe and effective (GRASE). However, these products are sold under the definition of “Marketed Unapproved Drugs” as they have been in use for a long time but may lack the rigorous testing needed [12]. GRASE category I includes 22 UV filters that are routinely used in sunscreen products. TiO_2_ and ZnO are most used as mineral or physical UV blockers (Section 3.2, Table 1). Other currently marketed UV filters such as avobenzone, cinoxate, dioxybenzone, ensulizole, homosalate, meradimate, octocrylene, octinoxate, octisalate, oxybenzone, padimate O, and sulisobenzone are included in the GRASE category III, as they require further studies about their safety as topical agents (Section 3.2, Table 1).

It is important to note that concerning the general safety issues of chemical UV filters, FDA moved PABA and trolamine salicylate (organic sunscreen actives) from the GRASE category to the category “not safe for human use”.

In fact, all organic sunscreen active ingredients have limited or no data characterizing their absorption profile. Therefore, the FDA has advised the industry to conduct a variety of tests, namely, carcinogenicity and reproductive toxicity, before introducing them into the GRASE category [12].

All UV filters formulated as spray or powder must have their potential risks of inhalation and/or flammability rigorously evaluated, as there is a lack of toxicity data about them. The approval process from FDA is slower than the European process (EU Cosmetic Regulation (EC No.1223/2009) by the Scientific Committee on Consumers’ Safety). The reason is the fact that the FDA classifies new UV filters as over-the-counter drugs rather than cosmetics, as in Europe and other parts of the world. Accordingly, UV filters require extensive clinical data to be recognized as safe for use in humans [12].

## 4. The Next Generation of Sunscreens: Phytoactive UVA Filters

Current marketed sunscreen products present limitations associated with (a) the lack of customization of the color of the product to each natural skin tone, as the tinted varieties currently commercialized do not match all shades and skin tones, and (b) the damage to fabrics and clothing that sunscreens can provoke, namely, those which are tinted (can adhere to fabrics and may stain clothing) and have higher amounts of physical blockers, such as TiO_2_ and ZnO [12].

To overcome the limitations found, the cosmetic industry introduced an innovative sunscreen product, the powder brush-on, which is extremely useful for reapplication rather than primary UV protection. This product is applied easily and can be blended well on top of makeup. One disadvantage of powder brush-on sunscreen cosmetics is the addition of many chemical excipients that make the users more susceptible to irritant or allergic skin reactions, such as contact dermatitis. Excipients are used in all types of sunscreen cosmetics to make the product consistency smoother and more cosmetically acceptable to users [12]. Another problem of powder formulations is the potential risk of inhalation of their particles that may cause respiratory inflammation [12].

Concerning the synthetic UV filters used in sunscreen formulations, photosensitization reactions and photodegradation with the generation of oxidative free radicals causing cytotoxicity and genotoxicity are common related problems associated with conventional sunscreen products, as discussed earlier. A solution for these problems is the design of sunscreens containing natural components, especially natural UV filters. Preliminary research has been carried out in the recent years indicating that natural sunscreens are easily available and more economical than the synthetic ones [5]. Natural sunscreens are also more compatible with all skin types [5]. Plant materials capable of absorbing or blocking UV radiation have been extensively studied in the development of natural sunscreen products [56]. Among several photoactive components, phenolic compounds, such as flavonoids, are playing a leading role in this research as they can absorb in the UV range of 200 nm to 400 nm [13].

Considering the urgent need of improving the UVA protection of current marketed sunscreens, novel plant-derived UV filters with higher anti-UVA activity are desirable. Formulating a sunscreen with both photoprotection and antioxidant activity will generate a multifunctional product with significant benefits for skin health maintenance [57]. However, discovering new phytoactive molecules with this double functionality has been a challenge for the scientific community lately. Minimal features for natural UV filters are similar or higher UV protective activity compared with synthetic filters and free radical scavenger properties. The overall PFUVA must be significantly superior to the PFUVA present in current sunscreen preparations [58]. Natural UV filters may replace or reduce the number of synthetic molecules with similar functions [47]. They possess low UV absorption capacity compared with organic UV filters [59] because they act mainly through physical blockage. Their most useful property in a sunscreen is the excellent antioxidant action. In addition, plant-derived sunscreens are remarkable emollients, making the formulations more cosmetically attractive for application on skin. 

This article is focused on flavonoids with UVA protective activity that naturally occur in vegetables, fruits, grains, teas, flowers, and others. Flavonoids have variable phenolic structures that can be divided into various classes such as anthocyanins, catechins, flavanones, and flavones [14]. Flavonoids have been reported to have extensive biological properties, namely, anticancer, anti-inflammatory, and antioxidant [14,60]. As referred, this class of molecules is leading the research on the field of natural sunscreens and, consequently, examples of this research will be presented in the next sections. It is important to highlight that the research on UVA photoprotection is significantly less than the studies on UVB protection, especially focusing on a specific class of phytoactive molecules. Therefore, the literature search carried out retrieved a reduced number of molecules worthy to be described in this review. Additionally, the existence of research on flavonoid-loaded nanoparticles incorporated into sunscreens with enhanced antioxidant and UVA photoprotection was another criterion used to select the flavonoids to be described in this section, as the rationale designed for this review aimed to match both topics. Accordingly, the most relevant flavonoids are herein described. 

### 4.1. Quercetin and Rutin

Quercetin, a plant pigment, is the most ubiquitous flavonoid in nature [61]. Quercetin has antioxidant and anti-inflammatory properties, as well as the capacity to modulate several pathways within the cellular system [62,63,64,65]. Rutin is the glycoside form of quercetin, and it is highly abundant in plants and fruits (for example, buckwheat seeds, tangerine, orange, grapefruit, lemon, and lime). Rutin and other flavonoids are well known for their scavenging properties of reactive oxygen species and these antioxidant properties have been reported by many authors in in vitro studies [66,67,68,69,70]. Rutin is a non-toxic and non-oxidizable molecule, preventing a pro-oxidant effect in the human body [71].

Cefali et al. developed an O/W emulsion to be used as a sunscreen, containing a mixture of plant extracts enriched in flavonoids. The SPF, antioxidant activity, physicochemical stability, photostability, and skin permeation of flavonoids were determined. The formulation containing the mixture of plant extracts presented an SPF of 2.94 ± 0.4 using the Mansur method and a PFUVA of 2.4 ± 0.5 by the diffuse reflectance spectroscopy method. The product presented a ratio UVA/UVB of 0.78, confirming that the developed formulation showed the capacity for UVA and UVB protection [72,73]. It also exhibited antioxidant activity and UVA protection in in vitro assays, as well as photostability. The phytocosmetic was not an irritant to skin and rutin was found both in the stratum corneum and in the deeper epidermis, improving the antioxidant activity and sun protection effect of the sunscreen. Despite the low SPF value, the developed product is promising as a natural sunscreen, especially if associated with physical UV filters, which will increase its solar protection. 

Tomazelli et al. assessed the photoprotective potential of rutin by in vitro and in vivo methods, comparing sunscreen formulations containing 0.1% (*w*/*w*) rutin, 3.0% (*w*/*w*) avobenzone, and 8.0% (*w*/*w*) octyl dimethyl PABA, with a similar phytoactive-free formulation. In addition, skin compatibility and the antioxidant activity of rutin, in association with the referred organic UV filters, were investigated [74]. Peres et al. previously assessed the antioxidant potential of rutin by the DPPH (2,2-Diphenyl-1-Picrylhydrazyl) scavenging assay in a formulation containing 0.1% (*w*/*w*) rutin in individual association with 3.75% (*w*/*w*) ethylhexyl methoxycinnamate, 4.0% (*w*/*w*) octyl dimethyl PABA, and 10.0% (*w*/*w*) octocrylene. The findings of Peres et al. were compatible with the ones of Tomazelli et al., as rutin increased the scavenging activity of the formulation designed by Tomazelli et al. by 75%.

The results of Tomazelli et al. corroborated the work of Oliveira et al. in which rutin was associated with UVA filters in the DPPH assay. The authors used 0.1% (*w*/*w*) rutin in association with 6.0% (*w*/*w*) benzophenone-3 or 3.0% (*w*/*w*) butylmethoxydibenzoylmethane and observed that rutin raised about 40% of its free radical scavenging potential. 

According to all the results cited, rutin is an excellent antioxidant and is compatible with UVA and UVB conventional filters in sunscreen formulations. In addition, rutin has proved to be photostable and safe for use.

### 4.2. Silymarin

Silymarin is a standardized extract of Silybum marianum seeds and it is one of the most studied polyphenolic blends for photoprotective activity. Many authors have documented the ability of silymarin and its major component, silybin, to reduce UVB-stimulated skin damage [75,76]. Svobodová et al. demonstrated that silymarin and silybin decreased UVA-stimulated damage to normal human dermal fibroblasts [77]. However, this property in less abundant components, such as the flavonolignans isosilybin, silychristin, silydianin, and 2,3-dehydrosilybin, has not been studied yet. All these molecules showed higher antioxidant potential compared with silybin [78] and, thereby, may provide photoprotection. Subsequently, Svobodová et al. evaluated if isosilybin, silychristin, silydianin, and 2,3-dehydrosilybin could prevent UVA-induced damage to normal human dermal fibroblasts and compared their efficacy and contribution to silymarin UVA-photoprotective global effect. The results suggested that all four silymarin molecules can protect the skin from UVA harmful effects and the most potent seemed to be 2,3-dehydrosilybin followed by silychristin. These two molecules had significant effects on most of the studied parameters in vitro: (a) UVA-cytoprotective effect; (b) elimination of oxidative stress; (c) prevention of glutathione depletion; (d) reduction of caspase 3 activity and, therefore, of apoptosis; (e) modulation of DNA single-strand breaks; (f) prevention of the production of carbonyl proteins; and (g) reduction in the secretion of matrix metalloproteinases and stress proteins as heat-shock proteins [47].

### 4.3. Pomegranate

The scientific literature has shown that pomegranate, Punica granatum L., is a fruit with remarkable properties. The high antioxidant activity of Punica granatum juice was already reported. The authors described the antioxidant activities of pomegranate extract, focusing their attention on the antioxidant properties of anthocyanidins, such as delphinidin, cyanidin, and pelargonidin, as H_2_O_2_ (hydrogen peroxide) scavengers [79]. More recently, the main components of the juice were considered as potential sunscreens, as well, due to their high absorption capacity in the UVA and UVB range [79], evidencing the current interest of the scientific community towards the potential of the use of pomegranate juice against skin aging and carcinogenesis.

### 4.4. Lignin

Lignin is a heterogeneous phenolic polymer found in the structure of woody plants and most terrestrial plants, and it is the product of the co-polymerization of three different phenylpropane monomers: p-coumaryl alcohol, coniferyl alcohol, and sinapyl alcohol [80].

The aromatic lignin polymer is considered a metabolic product of the process of adaptation of some of the most advanced source plants to environmental adverse conditions, playing essential roles as UV shields, antioxidants, and precursors for structural biopolymers [81]. However, before these plants developed the ability to synthesize lignin, it is believed that flavonoid compounds were responsible for protecting them from UV radiation. It has been demonstrated that flavonoids can chemically link to lignin polymers through a natural process known as lignification in cross-coupling reactions with monolignols, acting in the derived products as natural lignin monomers with SPF booster properties. Marketed lignin is a by-product of pulping or, to a lesser extent, of biorefinery processes [50]. During both procedures, the structure of lignin in the plant degrades, forming smaller molecules with new functional groups, namely, new and diverse chromophores. Technical lignin is obtained from the pulping process and contains UV chromophores, including quinones and methoxy-substituted phenoxy groups, which can be conjugated with double bonds or carbonyl groups, increasing, therefore, the absorption of UVA and UVB radiation. Among all technical lignins available, kraft lignin is the most abundant. Remarkably, it has a more potent antioxidant activity than the commercialized antioxidant butylated hydroxytoluene. Additionally, it exhibits low cytotoxicity towards healthy animal cells and promising antitumoral activity.

The natural SPF booster capacity, the high antioxidant potential, and the absence of toxicity in physiological conditions favor the inclusion of technical lignins in marketed or newly formulated sunscreens. Most probably, no additional antioxidants will be required in the sunscreen in the presence of technical lignins.

Focusing on industrial activity, the hazelnut and walnut crops industry generates many shells annually as a by-product and it has been increasing over the last 30 years, making this industry very attractive to obtain high-value commercial products. Currently, these types of solid residues are mainly used for low-value applications, such as solid fuel, despite their high lignin content (30–50%) [82]. Therefore, the high potential of agro-industrial residues as a lignin source, in association with the lack of characterization of this type of lignins comparatively with technical lignins and lignins from other agricultural wastes, motivated Gordobil et al. to investigate the antioxidant and SPF booster potential of lignin isolated from hazelnut and walnut shells. The authors also evaluated the cytotoxic action of isolated lignin towards murine fibroblast cell line 3T3 as an essential requirement for topical sunscreens. Isolated and purified lignins from hazelnut and walnut shells showed significant antioxidant and UV-absorbing activity for both lignin types, but the SPF provided by the sunscreen containing each of the lignins did not reach the necessary requirements established for the prevention of UV skin damage. Gordobil et al. also observed that murine fibroblasts were not significantly affected by the addition of lignins at 24 h of exposure, suggesting the absence of cytotoxicity in physiological conditions. The authors showed that a sunscreen formulation with lignins from hazelnut and walnut shells can be a promising natural solution for improved UVA photoprotection. Ecological studies performed suggest that natural bodies of water contain dissolved lignins and the ecosystem has adapted to them. Concerning human health risks, lignins have been found neutral or beneficial, as discussed [53,83,84].

The results described indicate that several types of lignins are attractive potential substitutes, acting as SPF boosters, for synthetic UV filters in sunscreen products. In fact, the use of lignin in sunscreens has been reported. It has been suggested that the addition of lignin can increase the SPF of sunscreen lotions and provide broad-spectrum UV-absorbing and antioxidant properties. However, lignin derived from the pulping process is a dark heterogeneous material with minerals and organic impurities, which requires lignin modification, purification, and/or fractionation to improve sunscreens’ UV blocking performance and cosmeticity [85,86,87,88].

Lignin has an overall synergistic effect with sunscreen actives in commercialized lotions, increasing their UV filtering potential even after irradiation with UV rays. In one of the studies performed, diverse types of lignin were added to several commercial sunscreen products, in different amounts, and significant increments in UV absorbance were registered: adding 2% lignin to an SPF 15 sunscreen doubled its SPF to 30 and the addition of 10% kraft lignin increased the SPF to 50. Improved sunscreen performance after irradiation with UV rays was also reported: after 2 h of UV irradiation, the UV absorbance of a sunscreen containing 10% lignin increased by more than 40%. This result suggested the existence of a specific synergistic effect between lignin and other ingredients in sunscreen lotions, as well as the evidence of the antioxidant capacity of lignin [89].

In another study, where lignin microparticles were produced from organic acid lignin and tested as a UV absorber [90], a significant SPF increase in pure hand lotion from 1 to 3.53, with 5% lignin microparticles, was observed. Additionally, the UVA/UVB ratio obtained was 0.69–0.72, which indicates a good UVA protection. These results demonstrated that lignin microparticles have excellent antioxidant and UV protection capacities, being a natural alternative to synthetic UV filters in sunscreens, namely, to UVA filters.

Another study used a lignin derivative, lignosulfonate, which was selected to modify TiO_2_, a compound traditionally used as a physical or inorganic UV filter [91], to evaluate the ability of lignin to modulate the UV filtering activity of other UV actives. The results showed, firstly, that esterification occurred between the carboxyl groups of lignosulfonates and the hydroxyl groups on the surface of TiO_2_, indicating the capacity of lignin to be a coating biopolymer. The TiO_2_ surface was coated with lignosulfonate, as described. This coating process with lignin improved the availability of TiO_2_ in the sunscreens and significantly boosted its UV-blocking ability. TiO_2_ coated with lignin nanocomposites was, secondly, incorporated into a pure hand cream and the sunscreen performance was studied using TiO_2_ as the control. The SPF values of the formulations evaluated, containing 5%, 10%, and 20% lignin on the TiO_2_ surface, were 16, 26 and 48, respectively. This study demonstrated that lignin can be chemically modified to increase the UV photoprotection capacity of other UV filters present in the same sunscreen product, enhancing the overall UV blocking effect. Therefore, lignin can function as a UV filter adjuvant in a sunscreen formulation.

Composites of alkali lignin (from a commercial source) and kraft lignin (from agribiomass) with ZnO nanoparticles were reported as UV blockers in a blend with a hand cream [88]. A 20% mixture of lignin with hand cream showed about 93% UVB blocking capacity, whereas commercialized ZnO nanoparticles showed 75–90% UV blocking in the entire range. The mixture of lignin and ZnO nanocomposites showed 100% blocking in the UVB range and 85–95% blocking in UVA range. The study suggested that the synergistic effect of lignin and ZnO nanoparticles played a key role in providing excellent UV blocking potential to the sunscreen formulation.

A sunscreen cream prepared with bagasse soda lignin and ZnO nanoparticles, mixed in a pure hand cream, is reported as a good sunscreen product [89]. The authors formulated different creams with 10% ZnO nanoparticles and 5%, 10%, and 15% soda lignin. Pure cream SPF was around 1.1 (10% absorbance). The addition of 15% soda lignin increased its absorbance up to 88% (SPF 8), which is the same as using 15% ZnO nanoparticles. However, combining 5% ZnO nanoparticles with 15% lignin increased the UV absorbance to 92% (SPF of 12.5).

## 5. Nanocarriers to Improve UVA Photoprotection

Sunburns are a common result of insufficient volume of sunscreen applied to the skin, infrequent reapplication after excessive sweating or perspiration, and swimming, or using expired or denatured sunscreen products such as those stored in a car glovebox all summer. The cosmetic acceptability of the sunscreen is a parameter of extreme importance for the user, since it determines the application mode on the skin, the permeation through the skin, the frequency of use, the age of the user (for children a spray is preferable, for example), among other criteria, influencing the overall usability of the sunscreen [12]. Although TiO_2_ and ZnO have long been used as physical or inorganic UV blockers in sunscreens, and are approved by FDA as GRASE UV filters, nanoparticulate sunscreen systems are recent. They are transparent on the skin compared with conventional formulations, making them more cosmetically attractive.

Nanoparticles, especially from TiO_2_, are often coated with other compounds to prevent or reduce photo-oxidative reactions and improve UV filtering. An example already described in the previous section is the case of TiO_2_ coated with lignin particles. Nanoparticles of TiO_2_ and ZnO should be assumed to have the same efficacy and toxicological profile in humans as the non-particulate forms, but data are not solid enough yet. Concerning the toxicological profile in other biological systems, nano-TiO_2_ was shown to affect algae [92] and nano-ZnO was more toxic to algae than ZnO [93,94]. Both types of nanoparticles can bioaccumulate on the surface of organisms, such as algae, where they can be toxic even without entering the cells [95].

Biochemical pathways activated by TiO_2_ nanoparticles on human cells have been demonstrated by many researchers such as Grande et al. [96], Nohynek et al. [97], Hansen et al. [98], Europa, 2007 [99], and Ze et al. [100], which led the International Agency for Research on Carcinogens to classify nanoTiO_2_ as a possible carcinogen when inhaled in high doses. Therefore, sunscreens in the form of sprays should be avoided, as mentioned earlier, due to the presence of this probable carcinogen. More authors have reported the toxicity induced by TiO_2_ and ZnO nanoparticles to human health, namely, to neural cells, including stem cells, and fibroblasts, generally, mainly in in vitro models [5].

As previously mentioned, flavonoids are phytoactive molecules that have been studied as potential sunscreen ingredients due to their remarkable reactive free radicals scavenging activity, significantly preventing UVA pro-oxidant effects on skin. Some flavonoids show good UVA filtering activity, additionally, which increases their photoprotection potential, making these molecules remarkably interesting targets of investigation. However, their poor water solubility limits their topical administration [15]. During the last decades, much research has been focused on the formulation of poorly soluble drugs. Currently, nearly 40% of drugs marketed are poorly soluble and even 60% of drugs that come from direct synthesis are poorly soluble [16]. Another disadvantage of raw phytoactives is related to the fact that their UV blocking ability is insufficient, and they cannot be relied upon alone to obtain significant UV protection [59].

Facing the limitations of natural UV actives, there is an urgent need of defining strategies for the preparation of sunscreen products with higher UVA/UVB ratio and excellent antioxidant activity against UVA damage, together with an adequate topical biodistribution kinetics. One of the most well-studied strategies is the development of nanotechnological formulations [17] of combining plant-derived actives with significant UVA protection with synthetic UV filters [101,102]. 

Before discussing the novel flavonoids-loaded nanoparticle systems for UVA protection in sunscreen formulations, it is important to understand the structure of the skin (Section 2) and the general composition of nanoparticles in order to design efficient nanosystems suitable for retention in the most superficial layers of the skin. 

The knowledge of the constituents naturally found in human skin, such as the lipidic content of the epidermis, have allowed the development of topical biocompatible nanoformulations, which can form, ideally, an impermeable film against UVA radiation at the surface of the stratum corneum. There, the nanoparticles can release the flavonoid content to exert their photoprotection effect at the epidermis. 

**Figure 4:**
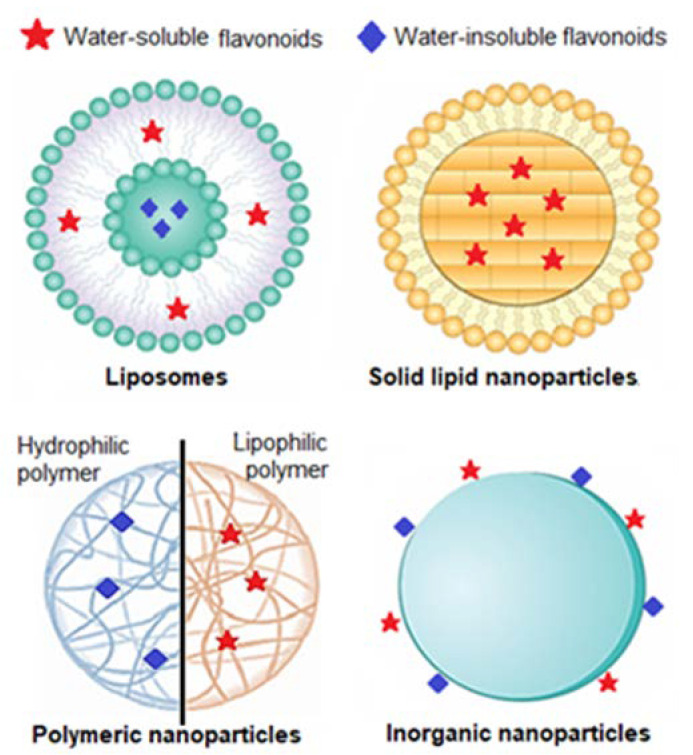
Types of nanoparticles mostly used for topical administration of UV filters on sunscreens *Adapted from* [103].

Taking this rationale into consideration, researchers have developed diverse types of nanosystems, with the most suitable for topical delivery of sunscreen natural UV actives being liposomes, solid lipid nanocarriers, polymeric nanoparticles, and gold nanoparticles (Figure 4). 

### 5.1. Liposomes

Briefly, liposomes were the first nanoparticulate systems to be studied as an effective delivery system through the skin [104]. They are biodegradable lipid vesicles composed of one or multiple lipid bilayers containing mixtures of phosphatidylcholines with long or short hydrocarbon chains.

The skin permeation of liposomes is dependent on their lipid composition (qualitative and quantitative), size, and surface charge. Concerning the surface charge, the positive charge of liposomes is helpful for the binding to negatively charged skin cells [105,106]. Concerning the composition, liposomes made from a more rigid lipid bilayer have a less efficient penetration profile, while liposomes made from a less rigid lipid bilayer have a better penetration profile and can penetrate the small epidermal–dermal junctions due to their deformable shape [107].

### 5.2. Solid Lipid Nanoparticles

These nanocarriers contain lipids that are solid at room temperature and whose surface is covered by a surfactant shell that stabilizes the dispersion. Solid lipid nanoparticles have gained more importance due to their uniform size, reduced surface area, and high drug loading capacity [108]. The application of solid lipid nanoparticles in the field of topical formulations improves the therapeutic efficacy by maintaining a controlled and sustained drug release and protects the drug if it is less stable. These nanoparticles can be used in both organic and inorganic sunscreen formulations [109]. A significant advantage of solid lipid nanoparticles is their very low toxicity, as the materials used in their preparation are biocompatible and biodegradable [110]. 

Concerning the interaction with skin components, previous studies suggested that solid lipid nanoparticles can penetrate through junctions of corneocytes and accumulate for several hours, allowing, consequently, the release of the encapsulated drug in a controlled and prolonged manner [109,111]. They have the capacity for bio adhesion and form a monolayer on the skin when the particle size is less than 100–200 nm. Since the monolayer film is hydrophobic, it has an occlusive action and, consequently, delays the loss of skin water, which can result in reduction of corneocyte packing and opening of inter-corneocyte gaps, with an improvement in drug permeation [112].

### 5.3. Polymeric Nanoparticles

Polymeric nanoparticles are usually more stable in vivo compared with liposomes and solid lipid nanoparticles. Due to their considerably large size, polymeric micelles can be used to co-deliver two or more UV filters for combinational sun protection. Due to their rigid and non-lipidic surface, polymeric nanoparticles are only able to penetrate the superficial layers of the stratum corneum and, from there, the encapsulated payload will be released into the deeper skin layers. 

The polymers used to produce these nanocarriers can be of natural or synthetic origin. One of the best studied natural polymers is chitosan. It is a biocompatible cationic polysaccharide extracted from crustacean shells and is capable of efficient drug delivery. In acidic conditions, the amino groups are protonated, conferring a positive charge to nanoparticles. The positive surface charge attracts the negatively charged skin molecules, as mucoproteins, increasing the bio adhesion to the skin surface [113].

Due to their polymeric nature, chitosan nanoparticles present two major advantages: a) sustained release of the encapsulated payload from days to months, and b) delivery of the payload through a pulsatile way, where release is dependent on the physiological needs of the person, minimizing the toxicity of the payload. The drug delivery mechanism is based on the degradation of chitosan, which depends on its molecular weight and degree of deacetylation. Therefore, the specific composition of chitosan is essential to define the release profile of the payload [113].

One of the most well-known synthetic polymers is poly (lactic-co-glycolic) acid (PLGA) [114]. PLGA has excellent biocompatibility and degrades through the physiological pathways. It has been tested for several medical and pharmaceutical purposes because it is approved by the FDA. This hydrophobic polymer can encapsulate a wide range of drugs, from hydrophilic to lipophilic, and benefits from the easy surface modification with specific ligands. It can be combined with natural polymers, such as chitosan, to deliver different payloads [115].

### 5.4. Gold Nanoparticles

Briefly, gold nanoparticles have the distinctive feature of reflecting and refracting UV light, according to their size, in a way that they can remain invisible in the skin. They act by an optical mode. The size of gold nanoparticles can be optimized to be large enough to reflect and refract the high energy of UVA and UVB radiation. Gold nanoparticles can be coated with bioactive ingredients of natural origin, for example, being converted into multifunctionalized nano systems with a preventive or therapeutic activity [116].

## 6. Phytoactive Nanoformulations with UVA Photoprotection Activity

**Figure 5:**
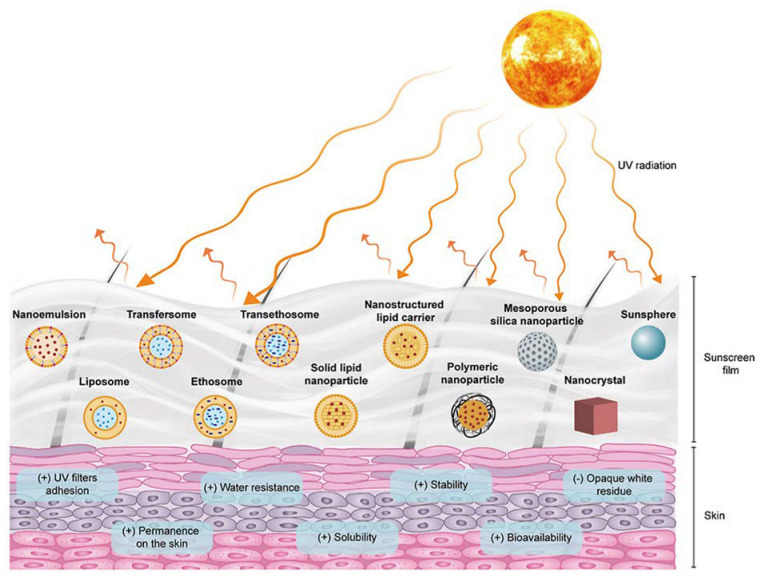
Multifunctional role of flavonoid-loaded nanoparticles in sunscreen products. *Reprinted with permission from* [117].

In this section, the research performed about the flavonoids described in Section 4 is presented, in which flavonoids were incorporated into nanoparticles in sunscreens, with enhanced anti-UVA activity (Figure 5). Once again, there are few studies on this topic; therefore, it is highlighted in almost all studies retrieved from PubMed. 

### 6.1. Quercetin-Loaded Nanoparticles

Among the most promising approaches to increase the bioavailability of quercetin through topical administration, lipid-based nanoparticles appear as the most attractive. Quercetin has been previously included in liposomes which lead to a 3.8-fold increase in penetration into skin than the aqueous suspension of quercetin [118]. Quercetin is also developed as second-generation nanocrystals, also known as smartCrystals^®^ technology [119,120] and lipid nano capsules [121,122]. Therefore, Hatahet and his coworkers decided to compare the efficiency of the three systems. Lipid nano capsules exhibited the smallest size of 26 nm, whereas the smallest liposomes were 179 nm and smart Crystals^®^ were 295 nm in size. Appreciable drug loading was exhibited by smart Crystals^®^ (14.4 mg/mL) and lipid nano capsules (10.8 mg/mL) where drug loading in liposomes was 0.58 mg/mL only. In the skin penetration studies, these nanoparticle systems demonstrated variable behavior. All formulations preserved free radical scavenging activity at 5 µg/mL of quercetin dose [123].

The in vivo skin penetration assay was performed with lipid nano capsules and smart Crystals^®^ to evaluate the release profile of quercetin. Liposomes were not included in this test because despite their significant antioxidant ability, they presented a very low loading capacity (0.56 mg/mL) and a large particle size (179 nm), compared with the other two formulations, which were not appropriate physicochemical properties for an efficient biodistribution through the skin. The higher skin penetration ability observed with lipid nano capsules compared with smart Crystals^®^ may be attributed to their lipophilic composition and the lower particle size (26 nm vs. 203 nm). Considering the hydrophilic composition of smart Crystals^®^ and their negative surface charge, these features make them inappropriate nanocarriers for deep skin penetration [120]. As a result, the superficial skin deposition of quercetin released from the smart Crystals^®^ nanoformulation favors their incorporation into sunscreen products. Authors concluded that quercetin-loaded smart Crystals^®^ may be a superior sunscreen, whereas quercetin-loaded lipid nano capsules may offer additional benefits of anti-inflammatory effects [123]. Quercetin-loaded large lipid nanoparticles may not be as efficient to deliver quercetin to the dermis and enhance penetration. For example, a comparative study showed that lipid nanoparticles (527 nm) exhibited skin penetration similar to the standard emulsion or an emulsion with a penetration enhancer [124]. 

### 6.2. Rutin-Loaded Nanoparticles 

Macedo et al. developed a stable rutin nano emulsion with a small mean particle size of 127 nm, a narrow polydispersity index of 0.168, and a zeta potential value near a neutrality of 3.49 mV. Despite the low zeta potential value, the stability of the nano emulsion was achieved, since the emulsion was sterically stabilized by soya lecithin and Tween 80 [125], preventing aggregation. The release of rutin from the nano emulsion was initially fast and then slower during the remaining time. This study showed that rutin can be successfully incorporated into a nano emulsion, which provides a controlled and prolonged release of the flavonoid over time [16].

Tomazelli et al. produced polymeric chitosan/tripolyphosphate nanoparticles loaded with flavonoids-enriched vegetable extracts, with an encapsulation efficiency of 75.89% of rutin. The polymeric nanoparticles exhibited a UVA/UVB value of 0.69, which suggested that the developed nanoformulation can be used as a UV filter for increased UVA protection. In addition, the photostability study was performed and, in the presence of the nanoparticles irradiated with UVA rays, the PFUVA value of the formulation was 2.0. The developed nanoformulation exhibited photostability, allowing the release of flavonoids from nanoparticles and the retention of rutin in the skin in a higher extension compared with the non-nanoparticulated form of rutin [126].

Another type of polymeric nanoparticles, i.e., rutin-loaded gelatin protein nanoparticles, were designed by Oliveira et al. [127]. The nanoencapsulation of rutin increased its antioxidant activity by 74% compared with free rutin. No decrease in the antioxidant activity was observed after UV irradiation, indicating a photostable profile for both nano-encapsulated and free rutin. Non-encapsulated gelatin nanoparticles, which were used as the control, exhibited an antioxidant profile as well due to gelatin amino acid residues. The association of rutin with gelatin, in a biodegradable nanostructure, had a synergistic effect on the antioxidant capacity of these components. Ethylhexyldimethyl, para-aminobenzoic acid (PABA), ethylhexyl methoxycinnamate, and methoxydibenzoylmethane were the synthetic UVB filters selected to be incorporated into the formulations to achieve a broad-spectrum profile. As they absorb UV radiation, they can photodegrade and generate free radicals. Methoxydibenzoylmethane reaches significant degradation with potential molecular loss of 50–90% after one hour of UV exposure. As referred above, rutin nanoparticles and free rutin exhibited a photostable profile as no decrease in the antioxidant activity was observed after UV irradiation. Consequently, the addition of rutin will not only increase the UVA protection, but also reduce the photodegradation of the synthetic UVB filters, thus improving the overall UV protection of the formulation. The in vitro photoprotective efficacy assay revealed an interesting profile for rutin nanoparticles. The UVA/UVB ratio obtained was 0.78, suggesting an improvement in the UVA blocking property. It is noteworthy that rutin nanoparticles had superior absorbance values in comparison with free rutin at the UVA and UVB range, which could be explained by an enhanced adherence of the nanoparticles on the surface of the skin, forming a protective film that could reflect and scatter the UV radiation. The rutin-loaded gelatin nanoparticles had good biocompatibility like free rutin. After 24 h of skin contact, no skin irritation such as stratum corneum disruption and no inflammatory reactions were observed.

### 6.3. Silymarin-Loaded Nanoparticles

Netto et al. produced solid lipid nanoparticles to deliver silymarin through the skin epidermis. The solid lipid nanoparticles were prepared by a nano emulsification technique, using the glyceryl monostearate as a lipid and Tween 80 as the emulsifier. The solid lipid nanoparticles were evaluated for silymarin entrapment, particle size and morphology, zeta potential, and polydispersity index. The nano emulsion prepared was incorporated in a sunscreen cream and several parameters were evaluated such as extrudability, viscosity, spreadability, silymarin content, in vitro silymarin release, ex vivo permeation of silymarin, in vitro and in vivo SPF determination, in vivo skin irritation test, and accelerated stability studies.

The authors suggested that as the concentration of the emulsifier increased, the loading efficiency of silymarin increased as well. The in vitro and in vivo SPF determination showed an SPF of 13.80 and 14.10, respectively. The stability studies were performed under accelerated conditions, and they did not show any significant change in the parameters analyzed. The in vitro and in vivo SPF values demonstrated that prepared sunscreen with silymarin-loaded solid lipid nanoparticles have excellent UV photoprotective action and can be a promising natural alternative for UV protection [128]. 

Concerning the major components of silymarin, silybin, isosilybin, silychristin, silydianin, and 2,3-dehydrosilybin, there were no studies focused on their nanoencapsulation in sunscreens found over the period of literature search defined.

### 6.4. Pomegranate-Loaded Nanoparticles

Gubitosa et al. focused their investigation towards the antioxidant activity of Punica granatum juice and its ability to screen UV radiation. They showed the eco-friendly formation of pomegranate-juice-induced AuNPs (gold nanoparticles), as a novel and promising efficient nano system combining the antioxidant and sunscreen properties of pomegranate with the biomedical properties of gold nanoparticles. The obtained nanocarriers are presented as a booster in sunscreen products, to improve their antioxidant activity and SPF values [79]. 

The plant-based synthesis of AuNPs has gained importance due to its low cost, high reproducibility, and eco-friendliness. Gubitosa et al. demonstrated that pomegranate juice used without further purification easily forms homogeneously coated AuNPs. The phenols from pomegranate are the chemical molecules involved in the AuNP formation reaction, leading to the bond of pomegranate chromophores to the surface of nanoparticles. The resulting capping, made of pomegranate functional groups, scavenged the free oxidative species, and filtered the UV radiation. Coated gold nanoparticles were obtained in a reasonable time, at least 4 h, using an appropriate amount of chloroauric acid. The acid pH value of the juice (pH 3) turned out to be the optimal condition for the synthesis because at pH values greater than 3, observed during the synthesis, aggregated nanoparticles were produced. The synthesized pomegranate-coated gold nanoparticles were stable in time and under extreme conditions of pH values and temperatures. They showed photostability, as well, under UV irradiation. The possibility of using antioxidant pomegranate-coated AuNPs as boosters in sunscreen formulations, exhibiting an SPF value of 6, was demonstrated, according to Gubitosa et al. [79].

### 6.5. Lignin-Loaded Nanoparticles

Strategies to increase the UV performance of pure lignin polymer are still needed, as discussed previously, to obtain successful lignin-based sunscreens. The dark color of lignin is the biggest limitation to the market promotion of lignin-based sunscreens [129,130]. 

When 5% lignin nanoparticles were added to marketed sunscreens, the SPF increased from 5.4 to 30.0, which suggested that the size of lignin particles is an important parameter to consider when formulating a sunscreen. Interestingly, bulk lignin absorbs only one-fourth of UV radiation when compared with lignin nanoparticles, demonstrating the importance of the smaller size of nanoparticulated lignin in its UV-absorption properties [89].

Recently, Lee et al. extracted lignin under mild conditions (at room temperature with neutral solvents) and incorporated the obtained light-colored lignin into sunscreens [131]. Cellulolytic enzyme lignin nanoparticles were produced using a solvent-shifting method combined with ultrasonication [132,133]. A cream mixed with 5% lignin nanoparticles presented SPF and PFUVA values about twice as high as those presented by the same cream mixed with cellulolytic enzyme lignin only. In addition, Lee et al. suggested that lignin nanoparticles had synergistic effects with an organic UV-filter sunscreen. The addition of 5% nanoparticles increased the SPF and PFUVA values of the sunscreen about 5-fold overall. The organic UV filters used were aromatic compounds such as octocrylene, ethylhexyl salicylate, butyl methoxydibenzoylmethane, and ethylhexyl triazone. The synergy observed between natural and organic UV filters was explained by the aggregation between the aromatic rings of lignin and organic UV filters. The ZnO UV filter was also tested, and no synergism was observed between lignin nanoparticles and ZnO due to the absence of aromatic rings in the inorganic UV filters [131]. 

Considering the excellent synergic effect between lignin and organic sunscreens, Zhou et al. formulated lignin–polydopamine nano capsules for encapsulation of avobenzone and ethyl methoxycinnamate, well-recognized organic UV filters, by high-intensity ultrasound processing [81]. Polydopamine is known as the most important synthetic analog of melanin. Previous studies showed that polydopamine can form a supranuclear cap in human epidermal keratinocytes, mimicking the behavior of natural melanosomes and, therefore, providing a UV-blocking function. In addition to the high biocompatibility and excellent UV resistance, the catechol groups in polydopamine provide remarkable adhesion to inorganic and organic surfaces. The resulting nano capsules had a spherical shape and could avoid direct contact between organic UV filters and skin, providing waterproof resistance and preventing the penetration of organic UV filters through the skin and the absence of skin toxicity. When they contacted the skin, catechol groups formed hydrogen bonds with the amino groups on the skin surface, while quinone structures formed C-N bonds with other amino groups through Michael addition or Schiff base reaction. The chemical bonds formed were strong enough to produce a superficial film containing these lignin nanoparticles. The phenolic hydroxyl groups and catechol groups are chromophores that enhanced the UVA and UVB absorption. The quinone functional groups favored UVA absorption, specifically. It was found that the SPF value of the sunscreen containing 10% nano capsules reached 195.33, which was an extraordinary UV photoprotection measure. It was also suggested that the nano capsule-based sunscreen was photostable as it maintained a high SPF value for at least 8 h. The photostability of nano capsules is closely related to the free radical scavenging ability of lignin and lignin–polydopamine association. The phenolic hydroxyl groups could scavenge the free radicals timely to avoid the consequent oxidation-induced degradation of organic UV filters. Another important result is the absence of leakage of organic UV filters during the experiment, which ensures the safety of the formulation. In conclusion, Zhou et al. showed that lignin–polydopamine nano capsules had a noticeable skin bio adhesion ability and could prevent the penetration of avobenzone and ethyl methoxycinnamate, as well as be waterproof. The nanoparticles had excellent UV resistance, especially against UVA radiation, very good antioxidant capacity, and photostability. The biocompatibility test showed that they were not cytotoxic and facilitated cell repair and growth, additionally.

Li et al. produced nanoparticles of lignin extracted with organic acid. The nanoparticles were obtained by dialysis in water, which changed the morphology of lignin, increased its specific surface area, and increased the resistance of lignin to UV radiation. The organic acid lignin nanoparticles were used as UV blockers in sunscreen formulations [90]. The lignin nanoparticles remained stable when stored in darkness for 180 days. They had a spherical shape and a mean size inferior to 100 nm. The size of the nanoparticles was bigger than the mean thickness of the stratum corneum of the skin, thereby they could not penetrate the dermis, avoiding the potential cytotoxicity to the skin cells. The nanoparticles showed UVA and UVB absorption capacity and a UVA/UVB ratio in the range of 0.69–0.72, thus demonstrating an evident UVA filtering potential. Using 5% nanoparticles, the SPF value of the lotions incorporated with them increased from 2.80 to 3.53, which was considered a significant improvement.

Gutiérrez-Hernández et al. produced nanoparticles by using lignin and ZnO as UV active ingredients [134]. Lignin was obtained from A. tequilana Weber bagasse by soda and organosolv pulping, a typical by-product of the tequila industry. A total of 6 types of lignin nanoparticles were prepared, 3 for each type of lignin source—organosolv or soda pulping—using 18% or 27% of active formaldehyde and having the lignin nanoparticles produced without formaldehyde as the blank. Concerning the ZnO nanoparticles, their production was accomplished by direct precipitation using zinc nitrate and potassium hydroxide. Sunscreens were prepared by adding 5% ZnO nanoparticles to a diverse amount of lignin nanoparticles—5%, 10%, and 15%—in a cream vehicle. Sunscreens containing only ZnO nanoparticles and only lignin nanoparticles in the range 5–15% were also prepared and used as controls. These mixtures of nanoparticles resulted in an additive increase in the SPF in these formulations, especially with lignin from soda pulping. For example, in the case of organosolv lignin, the addition of 5% ZnO nanoparticles in the formulations increased the SPF factor by about 4–5 units, which is the SPF value obtained with ZnO nanoparticles formulation (5%) alone. In the case of soda lignin, the increase in SPF values was 1.5-fold the additive value alone (for example, 4.5 ZnO nanoparticles and 4.5 soda lignin, where the combination resulted in a SPF value of approximately 13). A total of 3 samples reached a UV protection superior to 90%, suggesting that the combination of both nanoparticles can be considered as broad spectrum and photoprotective. Additionally, the combinations showed elevated UVA/UVB ratios—0.70–0.95 range—which means that they have a strong anti-UVA protection. The photostability was evaluated, as well. After 3h, nearly 90% of the SPF value was maintained, showing sustained photoprotection. An advantage of the combination of ZnO nanoparticles with lignin nanoparticles is that it also provides a pleasant tone to the skin, mainly to the darker natural tones, since the sunscreens with ZnO nanoparticles are whitish [134]. Table 2 is a compilation of all the studies described about natural nanoformulated sunscreens with evident UVA photoprotection.

Clinical trials concerning the UVA photoprotection efficacy and safety of plant-derived natural molecules for human skin, incorporated or not into sunscreen nano systems, were searched at www.clinicaltrial.gov on July 2022. For each phytoactive molecule presented, a combination with the following keywords was investigated: “skin cancer”, “photodamaged skin”, “sun damaged skin”, “sunburn”, and “sunburn, erythema”. One clinical trial was found related to phytoactive pomegranate. In this study, the skin benefits on aging and inflammation of pomegranate extract and juice were evaluated through dietary supplementation. The clinical trial was completed; however, no results were published until now for pomegranate. 

In addition to more clinical research, the improvement of sunscreen nano systems based on plant-derived UV active ingredients also needs innovative strategies that target pre-clinical research. An issue of major concern is the impact of the use of nanomaterials in ecosystems and human health, as the material coating the nanoparticles has been studied very little [11].

To better understand this impact, research focused on the permeation profile of nanosized formulations in intact skin and photo-damaged skin must be conducted. Most likely, an injured or dysfunctional skin offers different conditions for the penetration of phytoactives and the secondary ingredients of the nanoformulation, which can favor or limit certain toxic events on the cells.

The use of alternative in vitro models is a promising approach to define more reproducible and accurate pre-clinical models, as is the case in human skin models. The standardized definition of the biological targets of sunscreen nanoformulations is another strategy to be implemented, as it is fundamental to describe the cellular and molecular mechanisms underlining the therapeutic and toxicological effects of nanoparticles on human organs. Another challenge will be the regular preparation of sunscreen nanoformulations with safer excipients, such as soy lecithin/egg lecithin, beeswax, carnauba wax, castor oil, gelatin, soybean phospholipids, cholesterol, cetyl palmitate, soy cholate, etc., to encapsulate more phytochemicals or plant extracts with biocompatible and biodegradable polymers. 

As human skin is an exposed surface, external stimuli such as heat, visible light, or UV light can be used to control drug release on the skin for protection and to treat inflammation [135]. This idea shows the possibility of activating drug release after UV exposure in order to protect the skin when it is most needed, allowing the drug to work as a biosensor of UV radiation. Huang et al. studied a “smart” sunscreen system for skin photoprotection by using ZnO nanoparticles loaded with acetyl-11-keto-β-boswellic acid, a plant-derived UV active molecule. The system was evaluated on skin keratinocytes to investigate the release of the phytoactive after UVA irradiation. Interestingly, the ZnO nanoparticles were able to interconvert between hydrophobic and hydrophilic states upon light and dark exposure, respectively, which enabled the controlled release of acetyl-11-keto-β-boswellic acid from the nanoparticles. This result suggests that the amount of UVA exposure may influence the surface charge of the nanoparticles, which, in turn, conditionate the adherence to the negative surface of skin cells at the epidermis; therefore, the rate of release of the encapsulated phytoactive might change according to the intensity of UVA exposure. Furthermore, as the authors showed, UVA irradiation favors a hydrophobic state on the nanoparticles, which leads to higher adherence to the surface of the skin, and therefore favors the release of the encapsulated drug in opposition to what is observed when there is no irradiation. In this situation, the phytoactive becomes available to exert its maximum UVA protection at its maximum dosage loaded when there is, effectively, an exposure to sunlight, thus avoiding its continuous release that might cause adverse events on the skin. The phytoactives studied have remarkable anti-inflammatory and antioxidant activity, and the nanoparticles had low cytotoxicity. These findings demonstrated the UVA-triggered phytoactive release from a nano system as a more efficient and safer UV skin protection strategy. Therefore, “smarter” natural nanosized sunscreens should be formulated in the future [135].

## 7. Conclusions and Future Perspectives

Skin exposure to harmful UV radiation is the main risk factor for skin hyperpigmentation, aging, and carcinogenesis. In fact, skin cancer has been increasing, lately, despite the simultaneous increase in the sale of sunscreens. 

A detailed analysis of the approved marketed sunscreens worldwide unraveled the insufficient UV photoprotection of sunscreens and the negative impact of their compositions on ecosystems and human health. The reasons for these limitations are: (a) the predominant presence of UVB filters and less UVA photoprotection, although UVA accounts for 95% of total UV radiation reaching human skin; (b) the development of oxidative burst on skin layers after sun exposure, not prevented by current UV filters, which is responsible for DNA, protein, and lipid damage; and (c) the toxic effects of synthetic UV filters, the main type used so far, derived from their chemical photo instability. 

There is an urgent need to produce broad-spectrum sunscreens with significantly improved UVA and free radicals’ protection, which demonstrate a feasible safety profile. Meeting this need is a key milestone in the coming years. To achieve this, many researchers will have to dedicate their efforts to the formulation of alternative sunscreens based on plant-derived actives, with UV blocking and free radical scavenging activities, delivered to superficial skin by nanosized carriers with an adequate skin permeation profile. 

Flavonoids—quercetin, lignin, pomegranate, among others—are one of the most studied families of phytoactives in this field, while liposomes, solid lipid nanoparticles, polymeric nanocarriers, and gold nanoparticles are the better characterized nano vehicles for topical administration of those flavonoids. Remarkably interesting and promising results have been obtained from this research, through in vivo, ex vivo, and in vitro experiments, demonstrating the remarkable potential of flavonoid-loaded nanoformulations to be incorporated into marketed sunscreens or de novo synthetized semi-solid sunscreens. Many challenges still exist in this field, as the ecological and human impact of nanomaterials is not yet completely understood. The clinical evaluation of the efficacy and safety of these new natural nanoformulations is still lacking and the need to explore more plant sources for UVA filters is real and urgent, as well as the posterior combination of UVA filters with conventional filters in “smart” nano sunscreens.

## Figures and Tables

**Table 1:** Conventional synthetic UV filters approved worldwide: type, UV range of activity, maximum amount authorized in sunscreens, FDA category, and risks associated.

UV Filter	Type	Spectrum Activity	Maximum % in Sunscreens	Approvals and Possible Complications
Avobenzone	Organic or chemical	UVA	3% U.S. 5% EU, AUS 10% JP	Non-GRASE III Photodegradation Photosensitization
Octinoxate	Organic or chemical	UVA UVB	7.5% U.S. 10% EU, AUS 20% JP	Non-GRASE III Photodegradation Endocrine-disruption potential Skin absorption Breast milk detection
Octocrylene	Organic or chemical	UVA UVB	10%—worldwide	Non-GRASE III Photosensitization Skin absorption Breast milk detection
Oxybenzone	Organic or chemical	UVA UVB	6% U.S. 10% EU, AUS 5% JP	Non-GRASE III Possible photocarcinogen Skin absorption Breast milk detection Endocrine-disruption potential
Ecamsule	Organic or chemical	UVA	3% U.S. 10% EU, AUS, JP	No GRASE rating
PABA	Organic or chemical	UVB		Non-GRASE II Banned in Europe Allergen, contact dermatitis Possible photocarcinogen
Trolamine salicylate	Organic or chemical	UVB	12% U.S., CA, AUS 2.5% EU	Non-GRASE II Skin absorption Salicylism risk
Titanium dioxide	Inorganic or physical	UVA UVB	25% U.S., EU, JP No limit—AUS	GRASE I
Zinc oxide	Inorganic or physical	UVA UVB	25% U.S., EU, JP No limit—AUS	GRASE I

Abbreviations: U.S. = United States, EU = Europe, AUS = Australia, JP = Japan, and CA = Canada; GRASE = “Generally Recognized as Safe and Effective”.

**Table 2:** Main findings in studies performed about flavonoid nano-formulated sunscreens with evident UVA photoprotection.

Flavonoid	Pharmaceutical Formulation	Physicochemical Stability	UV Filters	UV Protection	PFUVA	UVA:UVB Ratio	SPF	Antioxidant Activity	Photostability	Skin Permeation	Skin Adverse Reactions	Ref.
Rutin	O/W Emulsion	-	-	Present	2.4	0.78	2.94	Present	Present	Stratum corneum and deeper epidermis	None	[49]
	O/W Emulsion	-	Avo benzone and octyl dimethyl PABA	Present	-	-	70% increase compared with free rutin	40% increase compared with free rutin	-	-	None	[74]
	Nano Emulsion	127 nm 3.49 mV	-	-	-	-	-	-	-	-	-	[16]
	Chitosan/tripolyphosphate (TPP) NP loaded with flavonoids-enriched vegetable extracts	Encapsulation efficiency = 75.89%	-	Present	2.0	0.69	-	-	Present	-	-	[126]
	Rutin-loaded gelatin NP	Encapsulation efficiency = 51.8%	Ethyl hexyl dimethyl PABA, ethyl hexyl methoxycinnamate, methoxydibenzoyl methane	Present	-	0.78	-	74% increase compared with free rutin	Present	NP adherence onto skin surface, forming a protective film	None	[127]
Quercetin	Liposomes Lipid nano capsules Smart Crystals^®^	179 nm 26 nm 203 nm	-	Present	-	-	-	More pronounced effect with lipid nano capsule and liposome	-	Lower skin penetration with Smart Crystals^®^	None	[123]
Silymarin	Solid lipid NP	-	-	Present	-	-	13.80—in vitro 14.10—in vivo	-	Present	-	-	[128]
Pomegranate	Pomegranate-juice-coated gold NP	100 nm Stable under extreme conditions of pH value and temperature	-	Present	-	-	3–18	Present	Present		Absent at 1.80 × 10^−12^–3.60 × 10^−12^ M [AuNP]	[79]
Lignin	Cellulolytic enzyme lignin NP	Spherical shape	Octo crylene, ethyl hexyl salicylate, butyl methoxydiben zoylmethane, ethyl hexyl triazone	Present	Five-fold in crease relative to sun screen wi thout lignin nanoparticles	-	Five-fold increase allowed by the loaded NP	-	-	-	-	[133]
	Lignin-poly dopamine nano capsule	Spherical shape	Avo benzone, ethyl methoxycinna mate	Present	-	-	195.33	Present	Present	Stratum corneum	None	[81]
	Organic acid lignin nanoparticle	Spherical shape Size < 100 nm Stable in size after 180 days	-	Present	-	0.69–0.72	2.80–3.53-fold increase	Present	-	Stratum corneum	-	[90]
	Lignin nanoparticle (lignin obtained from *A. tequilana Weber* bagasse by soda and organosolv pulping)	-	Zinc oxide NP	Present	-	0.7–0.95	1.5-fold increase	-	Present	-	-	[134]

NP: Nanoparticles.

## Data Availability

Not applicable.

## Abbreviations and Acronyms

AuNPs—gold nanoparticles, AUS—Australia, CA—Canada, CBD—cyclo-butane dimmers, CHOP—C/EBP homologous protein, DNA—deoxyribonucleic acid, DPPH—2,2-Diphenyl-1-Picrylhydrazyl, EU—Europe, FDA—U.S. Food and Drug Administration, GRASE—Generally Recognized as Safe and Effective, GRP78—glucose-regulated protein 78, H_2_O_2_—hydrogen peroxide, JP—Japan, MMP1—matrix metalloproteinase 1, MMP3—matrix metalloproteinase 3, NF-Kb—nuclear factor kappa-light-chain-enhancer of activated B cells, NO—nitric oxide, O/W—oil in water, PABA—para-aminobenzoic acid, PFUVA—UVA Protection Factor, PLGA—poly-(lactic-co-glycolic) acid, RNA—ribonucleic acid, RNS—reactive nitrogen species, ROS—reactive oxygen species, SPF—Sun Protection Factor, TiO_2_—titanium dioxide, U.S.—United States, UV—ultraviolet, UVA—ultraviolet A, UVB—ultraviolet B, ZnO—zinc oxide.
